# Visual scoring of bone scintigraphy in ATTR-CM: insights from the TTRACK study

**DOI:** 10.1093/eschf/xvaf028

**Published:** 2026-01-08

**Authors:** Hendrea S A Tingen, Thomas M Gorter, Riemer H J A Slart

**Affiliations:** Department of Nuclear Medicine, University Medical Center Groningen, Groningen, The Netherlands; Department of Cardiology, University Medical Center Groningen, Groningen, The Netherlands; Department of Nuclear Medicine, University Medical Center Groningen, Groningen, The Netherlands; Biomedical Photonic Imaging Group, Faculty of Science and Technology, University of Twente, Enschede, The Netherlands

**Keywords:** Bone scintigraphy, ATTR-CM, Amyloidosis, Amyloid cardiomyopathy, Perugini grade, SPECT

Transthyretin (ATTR) amyloidosis is a progressive protein misfolding disease characterized by extracellular deposition of insoluble amyloid fibrils in multiple organs and tissues, including the myocardium, thereby causing heart failure.^1,2^ Bone scintigraphy is the cornerstone imaging modality to non-invasively diagnose ATTR amyloid cardiomyopathy (ATTR-CM), employing one of three bone-seeking radiotracers: technetium-99m-labelled 3,3-diphosphono-1,2-propanodicarboxylic acid ([^99m^Tc]Tc-DPD), technetium-99m-labelled hydroxymethylene diphosphonate ([^99m^Tc]Tc-HDP) and technetium-99m-labelled pyrophosphate ([^99m^Tc]Tc-PYP).^3,4^ The exact binding mechanism of myocardial radiotracer uptake remains incompletely elucidated. Proposed explanations include binding to microcalcifications adjacent to amyloid deposits or inter-action with metal ions and sulfhydryl groups within the amyloid fibrils.^5,6^

In this issue of the Journal, Garcia-Pavia *et al*. describe bone scintigraphy data of the TTRACK study. TTRACK was a multi-centre, non-interventional, cross-sectional epidemiological study that evaluated the prevalence and characteristics of ATTR-CM patients within a cohort of patients with hypertrophic cardiomyopathy of unknown aetiology. The study was conducted across 20 centres in 11 countries, yielding a total number of 766 patients. Participants underwent bone scintigraphy with or without single-photon emission computed tomography (SPECT). Cardiac uptake of the radiotracers was visually graded according to the Perugini scale, ranging from 0 (no cardiac radiotracer uptake) to 3 (radiotracer uptake greater than bones), by two nuclear medicine experts, one at each participating centre and a centralized independent expert reviewer.^7^ For the differentiation between ATTR-CM and light-chain (AL) amyloidosis, monoclonal protein testing and transthyretin gene sequencing were performed for patients with abnormal cardiac uptake (grade 1–3).

The present study by Garcia-Pavia *et al*. revealed a high level of agreement between locally assigned and centrally reviewed Perugini grades (κ = 0.84, 95% CI 0.81–0.88). Of the 58 discordant cases, only four represented clinically relevant discrepancies (grade 0/1 vs. grade 2/3) within the [^99m^Tc]Tc-HDP and [^99m^Tc]Tc-PYP cohorts. This high level of concordance is reassuring, as treatment eligibility depends on the distinction between grades <2 and ≥2, underscoring the importance of accurate grade assignment. Furthermore, given the rarity of ATTR amyloidosis, nuclear medicine physicians may encounter patients with this disease only a few times during their career, making a reproducible and reliable scoring system essential for ensuring diagnostic accuracy and appropriate therapeutic decision-making, particularly outside specialized amyloidosis centres.

Similarly, agreement between planar and single-photon emission computed tomography (SPECT) imaging was excellent (κ = 0.93, 95% CI: 0.86–1.00), with discordant grades observed only in three patients with [^99m^Tc]Tc-PYP scans who scored 0 on planar images and 1 on SPECT, which were not clinically relevant. Interestingly, all discordant results occurred in the smallest SPECT cohort ([^99m^Tc]Tc-PYP, *n* = 11), whereas larger SPECT cohorts with [^99m^Tc]Tc-DPD (*n* = 37) and [^99m^Tc]Tc-HDP (*n* = 27) showed no discordance, suggesting potential differences between radiotracers. Current guidelines recommend performing SPECT in all patients with suspected cardiac amyloidosis to confirm myocardial radiotracer uptake rather than blood-pool activity.^4^ However, in this study, only 75 of 766 participants underwent SPECT, as the study protocol was developed prior to these recommendations. Despite the results showing that planar and SPECT imaging often yield highly similar findings without evidence of blood-pool misinterpretation in this study, routine SPECT remains recommended to avoid potential false-positive interpretations. Additionally, recent studies on quantitative SPECT suggest that beyond confirming myocardial uptake, SPECT may allow precise three-dimensional assessment of myocardial radiotracer distribution and absolute uptake values, which could improve monitoring of disease progression or treatment response in the future.^8^ This potential adds another rationale for performing SPECT, even when it appears to be concordant with planar imaging.

Notably, discordances between SPECT and planar imaging, and between on-site and centrally reviewed Perugini grades, may be clinically relevant not only for grade 0/1 vs grade 2/3, but also for differences between grade 0 and 1. Although a definitive non-invasive diagnosis of ATTR-CM and subsequent initiation of therapy requires at least grade 2 cardiac radiotracer uptake on bone scintigraphy, additional diagnostic evaluation or prolonged follow-up may be justified in patients with grade 1 uptake if clinical suspicion for cardiac amyloidosis is high. In this study, among on-site vs centrally reviewed grades, 4 patients were scored as grade 1 on-site and grade 0 centrally, while 12 patients were graded 0 on-site and 1 centrally. Limited data exist on the characteristics and long-term outcomes of patients with grade 1 uptake. Some studies report a stable phenotype and favourable survival over long-term follow-up, whereas others show progression to grade 2 or 3 radiotracer uptake in some patients, and overall worse outcomes compared with patients without cardiac radiotracer uptake.^9,10^ As the prognostic and diagnostic meaning of grade 1 uptake is not well established, clinicians may reasonably pursue additional testing or follow-up in such cases. Consequently, discrepancies between grade 0 and 1 cannot be considered trivial.

It is important to note that the observations above mainly pertain to wild type ATTR (ATTRwt) amyloidosis, the most common form of ATTR amyloidosis presenting with heart failure in cardiology practice. In carriers of pathogenic transthyretin gene (*TTR)* variants (causing hereditary ATTR, ATTRv, amyloidosis), grade 1 cardiac uptake on bone scintigraphy more likely represents early disease rather than a false positive result and the distinction between grade 0 and 1 is therefore important in these individuals.^11^ However, *TTR* variant carriers are generally enrolled in screening programmes at specialized amyloidosis centres and are outside the scope of everyday cardiology practice.

Another key question addressed by Garcia-Pavia *et al*. is whether the three commonly used bone-avid radiotracers are equivalent in detecting ATTR-CM. In clinical practice, these radiotracers are assumed to be interchangeable, with the choice for a specific radiotracer largely dictated by geographic availability.^4,12^ Available evidence from diagnostic accuracy studies and smaller comparative cohorts suggests broadly similar diagnostic performance among [^99m^Tc]Tc-DPD, [^99m^Tc]Tc-PYP, and [^99m^Tc]Tc-HDP. Nonetheless, minor variations in the distribution of Perugini grades and semi-quantitative uptake have been observed across studies, hinting that subtle radiotracer-specific differences may exist.^13,14^ For instance, extracardiac radiotracer accumulation is rarely observed with [^99m^Tc]Tc-PYP, whereas [^99m^Tc]Tc-HDP appears most suitable for detecting extracardiac amyloid deposition.^4,12^ To date, only two small head-to-head studies have scanned patients with two radiotracers, both supporting overall equivalence, though minor variations in visual scoring were observed.^15,16^

In the study by Garcia-Pavia *et al*., [^99m^Tc]Tc-HDP scans more frequently showed higher Perugini grades, [^99m^Tc]Tc-PYP scans accounted for most grade 1 cases, and [^99m^Tc]Tc-DPD scans included the majority of grade 0 cases (*Table 1*). If radiotracers truly yield different Perugini grades, this could have important clinical consequences, as current guidelines require at least grade 2 uptake for a non-invasive ATTR-CM diagnosis. Patients imaged with [^99m^Tc]Tc-HDP may be more likely to meet this criterion and be considered for therapy, potentially leading to overtreatment, whereas those imaged with [^99m^Tc]Tc-DPD could be undertreated if disease severity is underestimated. Awareness of these potential radiotracer-specific differences is essential to ensure accurate grading and appropriate management of ATTR-CM.

**Table 1:** Distribution of Perugini grades by radiotracer in the TTRACK study

Perugini grade	[^99m^Tc]Tc-DPD (%)	[^99m^Tc]Tc-PYP (%)	[^99m^Tc]Tc-HDP (%)
**Grade 0**	79.8%	61.7%	58.7%
**Grade 1**	2.1%	27.7%	1.2%
**Grade 2**	0.3%	3.2%	8.7%
**Grade 3**	17.8%	7.4%	31.5%

It should be noted that the TTRACK study was not designed to compare different bone-avid radiotracers, and the observations described here represent an exploratory analysis. Differences in characteristics between the radiotracer cohorts cannot be ruled out and may partially explain the observed variations in Perugini grades. These limitations highlight the need for a dedicated, prospective head-to-head comparison of [^99m^Tc]Tc-DPD, [^99m^Tc]Tc-HDP, and [^99m^Tc]Tc-PYP to clarify whether subtle radiotracer-specific tendencies truly exist and their clinical significance. Such a study would be challenging to conduct due to concerns about additional radiation exposure, though advances in imaging technology and reconstruction methods may make it feasible in the future. Until such data are available, radiotracers should generally be considered comparable, but subtle differences should be considered when interpreting Perugini grades. Importantly, regardless of these potential differences between radiotracers, timely bone scintigraphy remains essential in patients with suspected ATTR-CM, as early diagnosis allows initiation of disease-modifying therapies that are most effective at early stages.^17,18^
